# Dental Amalgam Phase-Down—Status, Alternatives, Strategies and Preparedness for Implementation: A Review

**DOI:** 10.1155/ijod/6688410

**Published:** 2025-09-12

**Authors:** Bernina Kyale Kisumbi, Olivia Awino Osiro, Loice Warware Gathece, Susan Wanjiku Maina

**Affiliations:** Department of Dental Sciences, University of Nairobi Dental Hospital, Nairobi, Kenya

## Abstract

**Background:** Nearly a decade since the Minamata Convention on Mercury, the progress on the phase-down of dental amalgam is unclear. Regional and national governments were tasked to develop guidelines and phase-down plans because of variations in oral health statuses and usage of restorative materials. The phase-out date of mercury-added products and manufacturing processes, unless otherwise specified, was between 2020 and 2025. Although dental amalgam was one of the few products recommended for a phase-down, the effects of the control of the manufacture, importation and exportation of mercury-added products are imminent among the many consumers who set a phase-down date of 2030. Therefore, this review aimed to analyse the situation by presenting the status of the dental amalgam phase-down, available alternatives, strategies and preparedness for implementation.

**Methods:** A systematic literature search was conducted in three electronic databases (PubMed/MEDLINE, Google Scholar and Web of Science) using relevant keywords and combinations of Boolean terms. This was followed by a manual search of relevant references. The included studies were organized into emergent themes, forming the basis of the present review.

**Results:** A total of 71 articles were included for the qualitative review, the majority being cross-sectional surveys. Four themes were identified—current status of the dental amalgam phase-down, perceptions on dental amalgam alternatives, phase-down strategies and preparedness for the implementation as demonstrated by knowledge, skill, competencies, attitudes, curricula and policies.

**Conclusion:** Regions are at different levels of phasing down the use of dental amalgam, with varying experiences regarding alternative restorative materials. Notably, the teaching and placement of posterior composites is at an all-time high, and there is evidence of curricula changes denoting a paradigm shift towards preventive, minimal intervention and adhesive dentistry. Evidence-based global guidelines would be useful for a synergistic dental amalgam phase-down approach.

## 1. Background

The first severe undesirable effect of elemental mercury to humanity was experienced due to methylmercury poisoning in Minamata City, Kumamoto Prefecture, Japan, on 21 April 1956 [1]. It was caused by the discharge of chemical factory waste water containing mercury into the Minamata Bay and Siranui Sea, leading to the bioaccumulation of mercury in fish that was consumed by the local population [2]. The Minamata Convention on Mercury was negotiated and adopted by the United Nations Environment Program (UNEP), World Health Organization (WHO) and 128 plenipotentiaries in 2013. It became a legally binding treaty on 16 August 2017. The goal of the Minamata Convention on Mercury was to reduce anthropogenic emissions and releases of elemental mercury and mercury compounds to protect human life and preserve the environment by calling for a discontinuation of specified manufacturing processes that utilise mercury and mercury-added products by 2020 [3].

Dental restorative materials are used in the management of dental pain, to restore function and aesthetics and to prevent tooth loss due to dental caries. Dental amalgam is a restorative dental material containing approximately 40%–50% elemental mercury by weight in the form of silver–mercury (Ag_2_Hg_3_) and tin–mercury (Sn_2_Hg) [4]. In 2010, it was reported that dental amalgam waste accounted for 260–340 metric tonnes of anthropogenic mercury release globally, of which 20%–30% entered the solid waste stream [5]. The Convention recommended a phase-out of manufacturing processes that utilise mercury and mercury-added products unless otherwise specified, and the phase-down of dental amalgam. It outlined nine dental amalgam phase-down measures that were abridged to a three-thronged strategic intervention, namely: waste management; knowledge management; and, health systems strengthening as an integral part of an equitable and sustainable reduction of the use of dental amalgam [6]. An erratic dental amalgam phase-out may cause deterioration of oral health in vulnerable populations, further widening oral health inequalities globally due to the inaccessibility of alternative restorative materials and a lack of preparedness among dentists to effectively utilise them [7–10].

Dental amalgam has served as a posterior restorative material for more than 150 years due to its strength, easy handling profile, affordability and longevity [11–15]. The majority of dentists are proficient in the principles and techniques of utilising dental amalgam [16, 17]. Although dental amalgam is not directly associated with health hazards [15], the mercury constituent has often raised concerns [18, 19]. As stipulated in Article 4, paragraph 3 and Part II of Annex A of the Minamata Convention on Mercury, the dental amalgam phase-down entails a gradual reduction in the use of dental amalgam, while upholding best waste management practices, enhancing oral health promotion and prevention of dental caries, increasing the use of dental amalgam alternative restoratives (DAARs) for posterior teeth restorations and fortifying research on quality mercury-free alternative dental restoratives [3]. Dental professionals are key stakeholders in ensuring reorientation from traditional dental amalgam restorative philosophy to preventive and minimal intervention approaches, including demonstrating requisite competencies to execute the alternative restorations in posterior teeth [20, 21].

Notably, the challenges and strategies may differ across regions due to varied levels of dental caries disease burden, dental professional profiles, dental training curricula and oral health policy priority areas. Auspiciously, aesthetic demands from patients have led to a decrease in the use of dental amalgam in favour of tooth-coloured alternatives in both teaching and clinical practice thus promoting the phase-down globally [22]. Therefore, a multisectoral approach, including dental professionals, dental educators, governments, dental materials suppliers, dental insurances and patient representatives is necessary [7, 23]. From the experiences of forerunners, consultative engagements among the dental fraternity and contributions of other stakeholders resulted in the successful phase-out of dental amalgam in countries such as Norway, Denmark and Sweden [24]. Increasing the level of awareness, positive attitudes, commitment to change, aligning dental curricula and continuous professional development (CPD) of dentists who were trained prior to the advent of posterior alternative restorative materials will require policy agendas and guidelines to ease facilitation and implementation. Inclusion of the oral health agenda in Universal Health Coverage (UHC) and Primary Health Care (PHC) national strategic plans are avenues for prevention of oral diseases and promotion of oral health, which are key parameters for achieving the dental amalgam phase-down through reducing the need for dental restorations [6].

It has been approximately a decade since the adoption of the Minamata Convention on Mercury in 2013. Now boasting an impressive number of 152 parties who have committed to phase-out mercury-based manufacturing and mercury-added products [25], little has been documented about the achievements and progress regarding the phase-down of dental amalgam. Therefore, the aim of this review was to describe the global status of the dental amalgam phase-down and the perceptions on alternative restorative materials. Further, the review aimed to describe the phase-down implementation strategies as a paradigm shift from traditional restorative philosophies, and to establish the preparedness of the dental profession and other stakeholders for the same, as evidenced by their knowledge, skill, competencies and attitudes through evolving curricula and policies.

## 2. Methods

### 2.1. Search Strategy

A literature search of PubMed/MEDLINE, Google Scholar and Web of Science was conducted in March 2024 and repeated in February 2025 to identify all relevant studies published between 1 April 2000 and 30 April 2024, using various combinations of Boolean terms and MeSH terms of the following keywords: ‘dental amalgam', ‘dental amalgam phase-down', ‘Minamata Convention', ‘Minamata Convention on Mercury', ‘dental amalgam alternatives', ‘dental amalgam phase-down implementation', ‘dental amalgam phase-down approaches', ‘dental amalgam curriculum', ‘dental amalgam phase-down curriculum' and ‘dental amalgam phase-down policy'. The reference lists of the selected articles were also hand-searched for additional studies. Titles and abstracts of all identified articles were screened by two independent co-authors (Bernina Kyale Kisumbi and Olivia Awino Osiro), and irrelevant studies were excluded. Full texts of the potentially relevant studies were obtained and evaluated by the two authors; disagreements, if any, were resolved via consensus. There were no restrictions on language. All article types were included in the search.

### 2.2. Research Question

The addressed research question was: ‘What is the current status, alternatives, strategies and preparedness for implementation of the dental amalgam phase-down?'

### 2.3. Inclusion and Exclusion Criteria

**Figure 1:**
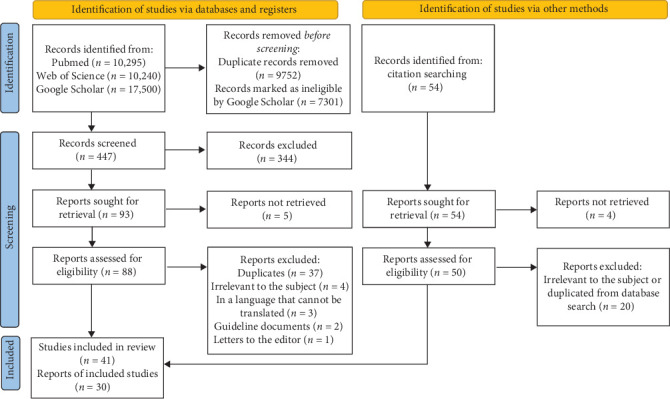
Flow chart of article identification, inclusion, exclusion and selection.

The inclusion criteria were articles that mentioned any aspect of the dental amalgam phase-down or alternatives to dental amalgam within the context of the phase-down. Thousands of articles were available on the topic of dental amalgam or mercury phase-out, but not within the scope of the phase-down, hence they were excluded (Figure 1). The exclusion criteria were: (i) inability to retrieve the full text, (ii) duplicates, (iii) articles that were irrelevant to the phase-down within the context of dental amalgam, (iv) articles in alternate languages that could not be translated to English to facilitate data extraction, (v) practice guideline documents and (vi) letters to the editor.

### 2.4. Article Selection and Data Extraction

Full texts of articles that seemed to fulfil the inclusion criteria were collected for further review; there were 88 from the electronic search and 50 from the manual search. The relevant information was extracted and summarised onto a spreadsheet by Bernina Kyale Kisumbi and verified by Olivia Awino Osiro. In total, 71 articles were included for the qualitative review—41 from the electronic search and 30 from the manual search—while 15 from the electronic search were excluded for reasons such as: inability to retrieve the full text, articles irrelevant to the phase-down within the context of dental amalgam, articles in alternate languages that could not be translated to English to facilitate data extraction, practice guideline documents and letters to the editor. Four articles from the manual search were excluded because they could not be retrieved. Further, duplicated articles were 37 from the electronic search and 20 from the manual search. For each selected article, the following data were extracted: authors, year and country of the study, study design, participants and the emergent themes.

### 2.5. General Characteristics of the Included Studies

There was a diverse global representation among the studies identified for review. The majority were cross-sectional survey studies targeting dentists, both general practitioners (23) and specialists (1), or dental educators, either heads of dental schools (5), heads of restorative dentistry (4), both heads of dental schools and restorative dentistry (2) or faculty (2); few involved dental students/interns (2), dental assistants (1), dental clinics (2) or patient records (2). The other common types of articles were reviews (9), opinions (7), and proceedings of meetings (6). There were also mixed-methods (2), qualitative (1), discrete choice experiment (1) and choice-based conjoint analysis studies (1).

Four themes were identified in the literature included as follows: current status of the dental amalgam phase-down globally, in high-income countries (HICs) and low- and middle-income countries (LMICs); perceptions on DAAR materials; dental amalgam phase-down strategies; and, preparedness for the implementation of the dental amalgam phase-down as demonstrated by knowledge, skill, competencies, attitudes, curricula and policies. The review is thus structured in line with these themes, and the corresponding literature is summarised in Table 1.

## 3. Discussion

### 3.1. Global Status of the Dental Amalgam Phase-Down

Globally, countries are at different levels of phasing down the use of dental amalgam even as the burden of untreated dental caries continues to pose a major public health challenge [22, 92]. The levels of dental disease burden, prevention, prioritisation and promotion of oral health and legislation on access and usage of dental materials also vary across various socioeconomic circumstances [7, 93].

From the articles reviewed, it was noted that the dental amalgam phase-down initially faced resistance from dental professionals due to lack of awareness, reluctance to invest in new equipment and varied competence levels in adhesive dentistry procedures, as observed in Norway [24]. In Australia, there was a sense of apathy and resignation [26]. Lessons from countries, such as Norway, that have successfully phased out dental amalgam corroborate the importance of a multisectoral approach. Evidence-based national plans generated through stakeholders' participation together with an overarching legislative framework to support the phase-down have been shown to streamline the implementation process [7]. In Australia, it was reported that dentists experienced challenges in placing DAARs, operator skill being a considerable factor in the success [27, 28].

Dental amalgam has been a significant component of restorative dentistry training curricula of dental schools in North America, Europe, Asia and Africa [29]. However, over the last two decades, the focus on dental amalgam has reduced, giving way to an emphasis on the teaching of posterior resin composites [30–42]. The trend reveals a gradual inclination towards the usage of dental resin composites for posterior restorations globally, positively reinforcing the endeavours of the dental amalgam phase-down.

#### 3.1.1. Dental Amalgam Phase-Down in HICs

Several HICs had restricted the use of dental amalgam prior to the global dental amalgam phase-down agenda. This was achieved through legislative strengthening or modification of policies on mercury, consultations within the dental fraternity, expert opinions on the feasibility of adoption of mercury-free restorations, media voicing of patient concerns regarding dental aesthetics and environmental factors [94]. These countries included Norway, Sweden, Denmark, Finland, the Netherlands, Spain, Mexico, Italy, Singapore, Austria, Canada, Japan, Germany, Bulgaria and the USA [24, 33, 43–45].

Norway, Sweden and Denmark implemented the ban on dental amalgam as a mercury-based product in 2008 [43, 45, 46, 94]. The usage has been markedly reduced in the Netherlands (1%), Finland (3%), Japan (4%), Germany and Switzerland (both 10%) [5, 24]. A recent study in Oceania dental schools reported that the preclinical teaching time of dental amalgam was 29% as compared to 39% for dental resin composites, whereas an average of 64% and 19.5% of posterior restorations placed were dental resin composites and dental amalgam, respectively, with a foreseeable phase-out of dental amalgam before 2030 [47]. A similar pattern was reported in Canadian [39] and American dental schools [40], where the preclinical restorative teaching time for dental amalgam had reduced to almost 25%, while that for posterior composite had increased to more than 50% between 2008 and 2018; furthermore, while more dental amalgam restorations were placed in the earlier timepoints, the recent trend showed a significant decline in the number of dental amalgam restorations and an increase in the posterior composite restorations being placed. An investigation on the use of restorative materials at the Faculty of Dentistry, University of Otago in New Zealand between 1998 and 2017 found that composite resin was the most commonly used material, followed by amalgam, glass ionomer and compomer in direct restorations. The use of amalgam decreased from 52.3% in 1998 to 7.1% in 2017, while a corresponding increase was observed in the use of tooth-coloured direct restorations, particularly composites [41]. Dentists and dental interns in Riyadh, Saudi Arabia, also reported reduced usage of dental amalgam, with 80.7% reporting that they did not use amalgam frequently. Moreover, dentists in private practice and dental interns were inclined to replace good amalgam restorations eliciting no complaints from patients with composites [42].

In Norway and four states in the United States—Maine, California, Connecticut and Vermont—patients are legally required to sign an informed consent document to receive a dental amalgam restoration [43, 46]. Nonetheless, reports still exist of dental amalgam use in developed countries. For example, in a recent study in the USA, 62% of general dentists and 56% of paediatric dentists were reported to be using dental amalgam [13]. In the UK, although there was a significant reduction in the use of dental amalgam compared with composites and glass ionomer cements (GICs), it still represented a large proportion (42%) of the nearly 1.8 million restorations placed in 2019 [48].

The European Union, through Regulation 2017/852 on Mercury (Article 10 [3]) mandated all members, including the UK at the time, to develop national dental amalgam phase-down plans, targeting a phase-out by 2030 [95]. This was amended through Regulation 2024/1849 in which, commencing January 2025, the export of dental amalgam was prohibited, and dental amalgam was not be used for dental treatment in the member states unless deemed strictly necessary by the dentist based on the specific medical needs of the patient. Furthermore, the import and manufacturing of dental amalgam was to be prohibited in the European Union [96] from July 2026. In HIC, government legislation, policies and funding have enhanced active engagement in research on alternative restorative dental materials, including long-term follow-up studies. Additionally, oral health promotion and disease prevention have led to a decline in the prevalence of dental caries, and consequently reduced the need for dental restorations, while duly considering the needs of their vulnerable populations [49, 95, 96].

#### 3.1.2. Dental Amalgam Phase-Down in LMICs

A few upper middle-income countries, such as Malaysia, China, Mongolia, Vietnam, Indonesia, Thailand and the Philippines, had also reduced the use of dental amalgam before the phase-down agenda [44]. On the contrary, lower middle-income and low-income countries are faced with unmet dental treatment needs, increasing prevalence of dental caries and a lack of dental public health policies and appropriate budget to support much-needed preventive strategies. Moreover, UHC and inclusion of the basic package of oral care (BPOC) within PHC is largely unrealised; therefore, restorative dental treatment is inaccessible to a large proportion of the population who are limited to dental extractions as the only affordable treatment [50, 51, 92]. Implementation of the dental amalgam phase-down is thus hindered by limitations of policy, funding and training.

The prevalence of dental caries is on the increase in developing countries [97] and the use of dental amalgam in posterior restorations remains evident in Abidjan (81.8%) [52], East Africa (49%–91.2%) [53–55], Nigeria (60%) [56], Iraq (57.8%) [51], Pakistan (87%) [66] and South India (57.3%) [57]. Nevertheless, the teaching and use of resin composites in posterior restorations globally has gradually increased over the last two decades due to aesthetic demand by patients and controversy of mercury in dental amalgam and the drive towards preventive and minimal intervention dentistry (MID) [30–33, 35–41]. This has also been reported in dental schools in South Africa [29] and among final-year dental students and dental interns in Kenya [34].

In Jordan, it was reported that the demand for aesthetic alternatives to dental amalgam by patients contributed to a reduction in the use of dental amalgam despite a low level of awareness of the Minamata Convention on Mercury and the dental amalgam phase-down. However, the study depicted variations in the use of dental amalgam, noting a marked decline in the private sector as compared to 43% in public facilities [58]. Likewise, in Pakistan, a reduction in the use of dental amalgam to 41.6% was reported, with 55% of respondents considering it a health risk; nonetheless, 76.5% were not familiar with the proper disposal protocols or whether relevant guidelines were available [59]. In Nigeria, discussions among stakeholders commenced, with calls for the government to review the national health care policy to include BPOC and MID in all the health programmes. Further, Nigeria phased out the use of dental amalgam in pregnant mothers and children under 16 years in 2020 [60–62].

Although Kenya was ratified as a party to the convention in 2023, there is a paucity of data on the dental amalgam phase-down. Two studies conducted among dentists in Nairobi in 2002 and 2010 showed that the preference for dental amalgam in posterior fillings was 50% and 76%, respectively [50, 54]. A study in East Africa (Kenya, Uganda and Tanzania) in 2012 involving 62 dentists during the initial phases of the dental amalgam phase-down agenda found a nearly equal proportion of the use of dental amalgam (91.2%) and dental resin composites (92.6%) for posterior restorations among the dentists [53]. However, a study on the selection of restorative and root filling materials among Kenyan dentists in 2015 found that the preferred direct restorative materials were resin composites (33.2%), GICs (30.3%) and dental amalgam (29.9%), while compomers had limited use (6.6%). In the permanent dentition, the use of resin composites was 52.6%, mostly for anterior restorations (30.3%), while dental amalgam was 49%, mostly for posterior restorations (89.5%). In the deciduous dentition, the use of GICs was 47.1%, while that of compomers was 21%. Overall, the site and type of cavity (22.5%) and material properties (19%) were considered during selection [55]. Moreover, an assessment of the status at the two dental schools in Kenya found that equal time was assigned to teach the principles of dental amalgam and alternative restorative materials, such as resin composites. Final-year dental students and dental interns expressed confidence in placing these alternative restorations and felt that the teaching received was adequate [34].

Reports from several LMICs also show a lack of best waste management practices which negates the aim of the dental amalgam phase-down. Dental amalgam scrap and waste is disposed of into general garbage, showing a general lack of adherence to recommended guidelines in countries such as Morocco (69.5%) and Burkina-Faso (49.6%) [63], East Africa (69.1%) [53, 64], Jordan (100%) [65], Pakistan (100%) [59, 66], Tunisia (94%) [67], Senegal (87.38%) [68] and Brazil (31.4%) [69]. Despite the challenges faced by LMIC, these studies demonstrate a commendable level of engagement on global trends by the dentists as key stakeholders, as they aim to promote oral health, disease prevention and provision of feasible and safe alternative restorations to their patients. Evidently, government support and funding are crucial yet limited elements for successful phase-down activities in these countries.

### 3.2. Perceptions on DAAR Materials

Increased demand for aesthetic restorations and measures proposed for the dental amalgam phase-down have resulted in concerted research efforts focused on improvement and development of DAAR materials that can match the strength, durability, packability and user-friendliness of dental amalgam [98–101]. Other restorative considerations include applicability in patients with high caries risk, poor oral hygiene, poor treatment compliance, large and subgingival restorations where moisture control may be challenging. Additionally, these alternatives seek to overcome the disadvantages of dental amalgam, namely, high biological cost during cavity preparation, lack of aesthetics and adhesive ability, low flexural strength, mercury content and resultant waste [82]. Presently, alternative restorative materials to dental amalgam are dental resin composites, polyacid modified composites or compomers, giomers, organic-modified ceramics (ormocers), high-viscosity GICs, resin-modified GICs, alkasites, dental ceramics, gold and stainless steel crowns [49, 99, 102–104].

#### 3.2.1. Overview of DAAR Materials

The versatility of composites for posterior restorations has been achieved through filler and resin technology to yield various categories [100, 105–107]. They include: bulk fill resin composites, which have reduced polymerisation shrinkage and propose a user-friendly one-stage incremental insertion, as the depth of cure is claimed to be 4–6 mm, similar to the condensing depth of dental amalgam [100]; organic modified ceramics (ormocers), which have been found to exhibit shorter clinical longevity than conventional resin composites but similar to that of bulk fill composites [108]; and, the novel bulk fill alkasites, a subgroup of resin composites which are suitable DAARs, as they demonstrate adequate flexural strength for use in posterior restorations and are also the only commercially available bioactive resin composite [99]. A comparison of the longevity of direct and indirect composites concluded that the two modes of application were similar in clinical performance on occlusal and proximal surfaces of posterior permanent teeth regardless of the material and tooth type [109].

GICs are believed to be biomimetic materials that are bioactive because of dynamic ionic exchange between the cement and tooth structure [110]. They have evolved considerably to yield materials with improved mechanical strength, moisture sensitivity and handling profile, while maintaining their salient features of chemical adhesion and fluoride release. Current materials include advanced or high viscosity GICs and zirconia-infused GICs [111, 112]. Several hybrid materials have also sprouted from the combination of GIC-based and resin composite-based materials to yield a continuum, including resin-modified GIC, pre-reacted glass (PRG) composite or giomer and polyacid-modified composite or compomer [113–115]. There is also a resin-modified GIC with a nanofilled resin coating (RC), the glass hybrids [103].

Currently, a myriad of high-strength dental ceramics are available, including glass ceramics such as leucite-reinforced, lithium disilicate-reinforced, glass interpenetrating composites like alumina and zirconia, and dense monolithic reinforced core materials such as pure magnesia, alumina and zirconia, applicable as full and partial coverage indirect restorations for posterior teeth [4, 104]. Dental ceramics are aesthetic indirect alternatives to dental amalgam and other cast alloys in large posterior restorations; however, they are expensive and difficult to process, thus comparatively more expensive for the patient [116, 117]. Additionally, a greater skill level is required and factors, such as poor oral hygiene, subgingival cavities, where moisture control is challenging and heavy occlusal loading may compromise the treatment outcome [104, 118].

Cast gold alloys are some of the most durable restorative materials with predictable clinical success in high stress-bearing areas in the arch and large restorations. However, it is the lack of aesthetics, chemical adhesion and material/high-technological fabrication costs that limit the use of cast gold restorations as dental amalgam alternatives [119–121].

Stainless steel crowns have been used in restoring grossly decayed deciduous molars, recording superior clinical performance over alternative direct restoratives like GICs, dental resin composites, giomers and compomers [122, 123]. Although there is limited information on prefabricated zirconia crowns for primary molar restorations [123], the clinical performance reported in the few available reports finds them comparable to stainless-steel crowns [124].

The articles reviewed on the DAAR dental materials presented various perspectives, including awareness, usage, efficacy, safety, performance and selection.

#### 3.2.2. Awareness of DAAR Materials Within the Context of the Phase-Down

Bailey et al. [70, 71] conducted two cross-sectional surveys in 2022. Regarding awareness of DAAR materials within the context of the phase-down, the first was to identify knowledge on the phase-down, opinions on a potential phase-out of amalgam and to assess the confidence of primary care clinicians in the UK using different materials in different situations. Respondents had limited knowledge of the phase-down and potential phase-out, and had major personal and patient-centred concerns, including lacking confidence in placing posterior composite restorations in difficult situations, unlike with amalgam. It concluded that effective education of clinicians and understanding patients' needs, alongside policy changes, were required to enable a successful amalgam phase-down and potential phase-out [70].

Makanjuola et al. [62] investigated the level of awareness and preparedness for the planned amalgam phase-down among Nigerian dental students and dentists and observed low levels of awareness regarding the Minamata Convention, amalgam phase-down, mercury hygiene practices and training in alternatives to amalgam among the majority of the respondents. Amalgam was commonly used by both dental students and dentists, yet only 4.7% of the respondents admitted to following good amalgam phase-down practices. The dental amalgam phase-down awareness and practices were significantly higher among dentists than dental students.

These studies revealed that the level of awareness on the issues around the phase-down and alternative materials was surrounded by uncertainties among certain populations of dentists, perhaps those who were trained much earlier than the year 2000. Therefore, in both HIC and LMIC, engagement and training of stakeholders to improve awareness and confidence is urgent and necessary.

#### 3.2.3. Usage of DAAR Materials Within the Context of the Phase-Down

As regards the usage of alternative restorative materials, Kopperud et al. [45] reported that 99.1% of Norwegian dentists were satisfied with composites for posterior restorations 1 year post the dental amalgam era. A practice-based study on US and Scandinavian dentists by Nascimento et al. [72] revealed that utilisation of composites was 48% in premolars and 49% in molars, while all other DAARs constituted 5%. The second survey by Bailey et al. [71] was to identify direct posterior restorative techniques, material use and reported postoperative complication incidence experienced by primary care clinicians in the UK. Amalgam use was found to be high in the publicly funded sector, and while composite was the most used alternative, it took longer to place, was more costly and associated with a higher reported incidence of postoperative complications. Although respondents were aware of techniques, such as sectional matrices to mitigate against food packing, they considered them time-consuming, thus their use was low. It was noted that major changes in health service structure and funding, and posterior composite education, were required in the UK and other countries during the phase-down.

These studies in HIC revealed increased uptake of composites as alternative restorations to dental amalgam; however, particularly in the UK, clinicians raised issues around cost, efficiency of treatment delivery and postoperative complications with composites. These are pertinent considerations where there is variation in funding for public and private oral healthcare provision, which may further influence the length of the appointment, hence the choice of the restorative material.

Umesi et al. [56] reviewed baseline data preceding implementation of the phase-down in Nigeria and found that amalgam fillings constituted nearly 60% of dental restorations. Emphasis was made on training in the placement of non-mercury alternatives to amalgam and MID, and a recommendation to intensify amalgam phase-down efforts. Alexander et al. [73] conducted a survey relating to the use of direct restorative materials among Australian dentists, considering the use of amalgam, year of primary dental qualification and association with academia to determine attitudes toward a phase-down of amalgam. Approximately one third (30%) of the respondents indicated they do not use amalgam, while the mean use of amalgam for all direct restorative procedures was 18%. Dentists were concerned over potential implications of a phase-down of amalgam yet undecided as to what they were, reflecting a lack of understanding of the issues. Alexander et al. [28] further conducted an online survey relating to aspects of and attitudes to the use of direct restorative materials among Australian dentists. The majority agreed that there was consistency in undergraduate teaching, but it was different from the 'real world.' Postgraduate education was ranked the most important influence on decision-making, emphasising the value of evidence-based dentistry. They concluded that clinical guidelines would be useful for standard practice.

These studies showed that the usage of alternative restorative materials was also high in Australia in the phase-down era despite uncertainties around the implications. Nonetheless, the need for evidence-based standard practice is relevant and timely.

An opinion article by Bayne et al. [74] on the challenge for innovation in direct restorative materials emphasised that the best way to avoid the use of dental amalgam is caries prevention due to limitations of amalgam alternatives. As such, material safety and environmental impacts are part of clinical risk assessment, and the focus for dentistry should be disease prevention and research on new alternative materials.

#### 3.2.4. Efficacy, Safety and Performance of DAAR Materials Within the Context of the Phase-Down

Regarding efficacy, safety and performance, a systematic review by Worthington et al. [75] sought to examine the effects (i.e., efficacy and safety) of direct composite resin fillings versus amalgam fillings in permanent teeth. To assess efficacy, they included randomised controlled trials (RCTs) that assessed restoration failure or survival at follow-up of at least 3 years, while to assess safety, they sought non-randomised studies in addition to RCTs that also measured toxicity, sensitivity, allergy or injury. They concluded that low-certainty evidence suggested that composite resin restorations may have almost double the failure rate of amalgam restorations, although the risk of restoration fracture did not seem higher with composite resin restorations; however, there was a much higher risk of developing secondary caries with composites. Further, very low-certainty evidence suggested that there may be no clinically important differences in the safety profile of amalgam and composites. Therefore, they maintained the support on the utility of amalgam restorations, particularly in parts of the world where it was still preferred to restore posterior teeth with proximal caries. However, they noted recent important improvements to composite resin materials, and in line with the global phase-down agenda, the choice of the restorative dental material will depend on shared decision-making between dental providers and patients in the clinic setting, and local directives and protocols. This review by Worthington et al. [75] was an update from the original by Alcaraz et al. [15] in 2014.

A systematic review by Kielbassa et al. [76] of highly viscous GIC/RC (hvGIC/RC) restorations reported that there were minor differences in failure rates between hvGIC/RC and GIC or composite resins as comparators in seven clinical studies. The hvGIC/RC combination showed high survival rates in occlusal cavities over 6 years. A follow up systematic review by Kielbassa et al. [77] on whether hvGIC/RC restorations (EQUIA Fil by GC) merged the Minamata Convention and MID concluded that within the respective indications and cavity geometries, the approach seemed promising and might be a restorative alternative for patients suffering from allergies or not willing to afford other sophisticated or expensive techniques. They also observed that the use of a light-cured nano-filled RC material may be advantageous in the short- and medium terms.

The above studies implied the safety and feasibility of composites, hvGIC/RC and GIC as alternative direct restoratives to dental amalgam. Within the recommendations of the phase-down, clinical decisions should be guided on a case-by-case basis in line with prevailing government directives to ensure quality patient care.

Aggarwal et al. [48] assessed the perceived impact of the post-Minamata amalgam phase-down on oral health inequalities in the UK through a mixed-methods investigation. Time–trends for amalgam placement showed a significant reduction in its use compared with composites and GICs. However, dental amalgam still represented a large proportion (42%) of the nearly 1.8 million restorations placed in the year of study. Some of the suggested direct impacts of a phase-down were related to increased costs and time to place alternative restorations and consequently, reduced quality of care, leading to increased tooth extractions, reduced access to care and privatisation of dental services, especially among deprived populations. They concluded that amalgam was still the preferred posterior restorative material in state run oral health services, and a complete phase-down would pose a threat to such services and widen oral health inequalities. Thus, a complete phase-out was not feasible at the time unless cheaper, long-lasting and easy-to-use alternatives were available and could be readily adopted by primary care oral health providers.

Despite the reduced usage of dental amalgam, the sentiments on cost and time implications of alternatives which may compromise quality and access to care by underprivileged populations were similar to those from the study by Bailey [70] in the UK. One way that the phase-down guidelines propose to address this issue is by discouraging dental insurers from varying the cost of dental amalgam and alternative restorative materials.

#### 3.2.5. Selection of DAAR Materials Within the Context of the Phase-Down

On the issue of selection, a qualitative study among Australian dentists by Alexander et al. [27] suggested that dentists' restorative decision-making was a complex interplay of ‘clinical', ‘knowledge', ‘patient', ‘practice type', ‘biological' and ‘environmental' factors. There was concern regarding the phase-down of dental amalgam yet there was a general sense of resignation or apathy to the matter.

A report by Austin et al. [78] on a debate by the British Society of Prosthodontics elicited specific concerns amongst clinicians regarding the suitability of mercury-free alternatives to amalgam; particularly where cavities are large and extend beneath the gingival anatomy. There were a few reports of lack of adequate undergraduate training on dental amalgam yet many clinicians, especially those treating patients for whom moisture control was challenging, felt that amalgam should remain available for certain clinical circumstances even in the event of a complete phase-down.

A concise review by Schmalz et al. [79] based on a FDI Policy Statement providing guidance on the selection of direct restorative materials as alternatives to amalgam recommended that ultimately, dental, oral and patient factors should be considered when choosing the best material for each individual case. Dental factors included the dentition, tooth type, and cavity class and extension; oral aspects comprised caries risk profiles and related risk factors; and patient-related aspects included systemic risks/medical conditions, such as allergies towards certain materials as well as compliance. Additionally, cost and reimbursement policies may need to be considered when amalgam alternatives are used, and the material recommendation will require the informed consent of the patient. The policy statement recommended using a patient-centred rather than purely a material-centred approach, concluding that further research is needed to improve overall material properties, the clinical performance, the impact on the environment, and cost-effectiveness of all alternative materials.

A discrete choice experiment through an online survey by Bailey et al. [80] to elicit the UK population's preferences for different attributes of restorations and their willingness to pay for restorative services and outcomes reported that overall, respondents were willing to pay to change the filling colour from silvery/grey to white and for increased restoration longevity. Ability to pay affected willingness to pay, with low-income respondents more likely to opt out of treatment and place less value on restoration colour (white) and increased longevity significantly as compared to those with higher income.

A cross-sectional survey by Al-Asmar et al. [58] to assess Jordanian dentists' perception and attitudes towards amalgam and composite restorations 4 years after the Minamata treaty was endorsed and suggest decision making factors that may influence the type of restoration requested by patients reported that Jordanian dentists used more composite restorations than amalgam. Recurrent caries followed by fracture of the restoration were the main reasons for replacement of both fillings by dentists. However, dentists suggested that the main reason patients requested replacement of amalgam was due to 'staining.' A large proportion of the dentists had experienced patients who had asked either for replacement of amalgam or refused an amalgam filling for aesthetic reasons; however, only 20% patients requested replacement of amalgam because of the mercury content. They concluded that the ‘phase-down' of dental amalgam was being implemented in Jordan's dental clinics but it was not associated with commitment to the Minamata Convention, rather to current dental practice trends and patients' aesthetic demands.

All these studies highlighted diverse patient, material and clinician factors that influence restorative decision-making, including material selection, and which will be applicable during the phase-down of dental amalgam in both HIC and LMIC.

### 3.3. Dental Amalgam Phase-Down Implementation Strategies

#### 3.3.1. Preventive, Minimally Invasive and Ultra Conservative Approaches for Dental Caries Management

The restorative model entails surgical removal of dental caries and material-driven geometric cavity extensions for retention and resistance of posterior dental amalgam restorations on premolar and molar cavities. In addition, this conventional restorative model is inefficacious at controlling the risk of future disease [125]. In pursuit of an evidence-based approach, restorative dentistry has experienced a paradigm shift towards a preventive and MID philosophy, whose common delineator is maximum preservation of healthy dental tissues, while employing adhesive alternative restorative materials [20, 126–128].

The concept includes caries risk assessment, caries prevention, non- and microinvasive approaches to control and reverse dental caries activity, delayed intervention, conservative tooth removal and replacement utilising the adhesive materials, preferably resin composite-based ones [21, 49, 129]. MID includes repair of imperfections of existing restorations among basic procedures, preserving the remnant intact restoration together with the sound hard dental tissues and involves caries management by risk assessment (CAMBRA) [77]. Further, it includes atraumatic restorative treatment (ART), which was originally developed for underserved communities but is now widely practised [20, 127]. An approach known as MID treatment planning (MIDTP) to implement MID in general dental practice has been described by Bassu et al. [130].

Dental caries prevention therapy includes caries risk assessment, advice to empower the patient with regard to caries risk factors, fluoride therapy, placement of fissure sealants and use of antibacterial mouthwash [131]. Overall, MID is a conceptual framework that commences with an all-inclusive diagnosis of caries, followed by primary prevention, management of the dental caries process and treatment of carious lesions by operative and non-operative approaches [132].

A study among dentists in public health practices in the USA found that those who had attended CPD training on MID believed that it met the standard of care for permanent teeth [133]. Another reported that younger dentists who had been trained on the MID strategies were likely to implement the same in practice; moreover, implementation of the new evidence-based concept in dental schools will precede its spread in general dental practice [129].

#### 3.3.2. Dental Amalgam Phase-Down Implementation Approaches

Four articles specifically addressed the phase-down approaches. Barkhuji et al. [81] employed a choice-based conjoint analysis to establish the strategic approaches to reduce amalgam use among active members of the American Academy of Pediatric Dentistry, specifically, the patient-related factors which influenced paediatric dentists' choice of amalgam. Responses from 45% who indicated using amalgam were analysed in the conjoint model. Selection of amalgam varied between 8% and 28% across all the clinical scenarios, with most respondents choosing composite or stainless-steel crowns. While both caries risk and type of insurance affected decision about amalgam use, caries risk was the driving factor in decision-making for using amalgam. The results underscored the importance of caries prevention and management of risk factors to successfully phase-down amalgam use. A systematic review by Kielbassa et al. [76] on whether hvGIC/RC restorations merged Minamata Convention and MID showed minor differences in failure rates between hvGIC/RC and GIC or composite resins. A follow up by Kielbassa et al. [77] concluded that within the respective indications and cavity geometries, the hvGIC/RC approach seemed promising, could merge the phase-down of mercury and the objectives of MID to some extent. These studies highlighted the clinical performance and feasibility of alternative restorative materials in minimally invasive approaches.

An opinion article by Mackey et al. [82] highlighted that while the Minamata Convention called for a voluntary phase-down of dental amalgam use and commitment to other measures, it fell short by failing to require binding and measurable targets to achieve these goals. They recommended that stakeholders within the international community should begin exploring ways to strengthen the implementation of the dental amalgam treaty provisions by establishing binding phase-down targets and milestones as well as exploring financing mechanisms to support treaty measures to ensure equitable access to global oral health treatment, while also promoting responsible environmental stewardship.

This has been implemented in the European Union through Regulation 2024/1849 [96] and the same approach could be adopted by other regions, making due considerations regarding unique socioeconomic circumstances and potential challenges to minimise oral healthcare inequalities. Evidence based decision making, minimally invasive dental treatment, and dental education should align with new conservative approaches.

### 3.4. Dental Amalgam Phase-Down Preparedness

#### 3.4.1. Dentists' Knowledge, Skill, Competency and Attitude

In a study by Al-Rabab'ah et al. [83] among dentists in Jordan to assess their knowledge of the phase-down of dental amalgam and their training and competency in placing posterior composites, out of 230 dentists, only 27 (13.8%) knew about the Minamata Convention on Mercury. Younger dentists were more likely to have received training related to the placement of DAARs, whereas almost half of those surveyed indicated that they would opt for CPD to improve their skills. In Kenya, a study among final-year dental students at two dental schools reported high levels of knowledge and high usage of dental amalgam alternatives [34].

In a study among Norwegian dentists 1 year after the ban of dental amalgam, a positive attitude and satisfaction with dental resin composite as an alternative to dental amalgam was reported; further, dentists chose minimally invasive treatment options to repair old amalgam restorations and also delayed operative intervention of early approximal lesions [45]. Alexander et al. [28] reported that among 408 graduating dental students, 91% indicated that they were competent at placing dental amalgam as opposed to 53% for dental resin composites, with the year of qualification playing a key role. Lynch and Wilson [23] reiterated that acquisition of skills to execute quality DAARs for dentists practising traditional operative dentistry would entail learning relevant scientific principles and artistry and capacity building with regard to knowledge, skill and understanding to manage different situations encountered in clinical practice.

These studies suggest that in both HIC and LMIC, dental students and dentists who were trained during the dental amalgam phase-down era acquired more knowledge and skill, as well as a more positive attitude regarding the use of alternatives to dental amalgam. Training efforts should therefore continue to emphasise on the agenda.

The attitude of dentists has been reported to affect the transition from use of dental amalgam in the traditional restorative philosophy to the preventive and minimal intervention care using alternative restorative materials for posterior teeth, which in turn can affect training to build the needed competencies [73, 84, 85]. In the study by Kopperud et al. [45], most dentists chose minimally invasive- or medium-invasive approaches when restoring fractured amalgam restorations. Additionally, they practiced delayed intervention of approximal carious lesions, which is a key component of MID.

#### 3.4.2. Dental Amalgam Phase-Down Curricula and Policy

The alignment of dental curricula to progressively teach more of dental caries prevention, MID and use of alternative restorative materials will equip the current and future dental workforce in preparing for the phase-down [29, 30, 32, 36, 86]. Dental caries prevention and the concept of MID as measures to phase-down dental amalgam are already being taught in modern core cariology curricula dental schools globally—Europe [87], Colombia [88], Spanish-speaking schools in Latin America [89], USA [90] and Oceania [47]. Nevertheless, there are variations in the implementation rate [129].

Bailey et al. [70] found that many primary care clinicians in the UK lacked confidence in using composite to restore posterior teeth in difficult situations as compared to amalgam. This necessitates effective education of clinicians and understanding patients' needs, alongside policy changes to enable a successful amalgam phase-down and potential phase-out. Likewise, Makanjuola et al. [62] reported low levels of training in the use of alternatives to amalgam among Nigerian dental students and dentists, while Umesi et al. [56] also emphasised the need for training of dental students in the placement of non-mercury alternatives to amalgam.

A mixed methods analysis by Alexander et al. [85] to ascertain dental educators' attitudes towards the teaching of dental amalgam at dental schools in Australia and identify preferred curricular approaches in a potentially 'amalgamless' profession concluded that there was a broad consensus of dental educators at Australian dental schools as how best to approach the teaching of amalgam within a phase-down. In Jordan, Al-Rababah et al. [83] concluded that dentists were not well informed on the Minamata Convention and the phase-down of amalgam, and more training in posterior composite placement was required in the undergraduate curriculum and continuous dental education for dentists.

These studies highlight the diversity in curricula and implementation policies and which are seemingly dependent on the study period in relation to the commencement of the phase-down agenda. Therefore, there is a need for harmonisation in phase-down policies and approaches in both HIC and LMIC.

An opinion article by Lynch and Wilson [23] to consider the educational and training issues in the present and future use of posterior composites highlighted ways in which dental school teaching and CPD may contribute to the successful phase-down, and inevitable discontinuation, in the use of dental amalgam. It underlined the need to embrace the use of alternate materials such as resin composites for restoring posterior teeth and highlighted the increased training need (undergraduate and CPD) in the application of posterior composites and other suitable materials for restoring posterior teeth.

Sidhu et al. [91] conducted a survey among faculty members to investigate the current and future teaching of posterior composite restorations in undergraduate curricula in Malaysian dental schools. All schools indicated the use of posterior composites for two- and three-surface cavities in premolars and molars. The didactic teaching time devoted to composites was greater than for amalgam, and most posterior restorations placed by students were composites, in slot-type cavities under mandatory rubber dam for moisture control. At the time, the phase-down of teaching and use of amalgam in Malaysia was expected to occur within the next 6 years although the use of amalgam was still taught. An online survey by Alexander et al. [28] among Australian dentists reported that there was consistency in undergraduate teaching although it was different from the 'real world.' Postgraduate education was the most important influencer of decision-making, highlighting the value of evidence-based dentistry and standardised clinical guidelines.

A survey on the teaching of posterior composites among dental schools in Oceania by Loch et al. [47] found that all respondent schools taught the use of posterior composites for occlusal and occluso–proximal cavities in premolars and molars, and the mean percentage of preclinical teaching devoted to composites was greater than for amalgam, anticipated to be 3:1 for posterior composite/amalgam in 5 years' time. The students were placing more composites than amalgam restorations, in slot-type cavities and under mandatory use of rubber dam for moisture control. The most common contraindication to composite placement was a history of adverse reaction to composites. The phase-down of teaching and use of amalgam in Oceania was expected to occur within 8–10 years. They concluded that despite minimally invasive approaches becoming increasingly common worldwide, the use of amalgam was still taught in Oceania. Future studies were necessary to assess whether the clinical teaching of posterior composites was in keeping with material development and trends in mainstream dental practice. At the University of Otago, it was noted that despite having no official phase-down policy, the Faculty of Dentistry had adopted the global trend to reduce the usage of amalgam although its teaching continued. It was recommended that curriculum changes were necessary to prepare graduates for clinical practice [41]. Likewise, the time dedicated to teaching amalgam restorations in Canadian and American dental schools was found to be high in comparison to the usage against posterior composites, thus necessitating curriculum review [39, 40].

These studies showed global efforts to adjust the curricula to increase clinical teaching time for dental amalgam alternatives with an inevitable reduction in training time spent for dental amalgam.

## 4. Conclusion and Recommendations

The discussion regarding the dental amalgam phase-down gained momentum in 2013 following the adoption of the Minamata Convention on Mercury. Prior to this, Scandinavian countries had banned the use of dental amalgam as a mercury-added product in 2008. The agenda was found to be active as evidenced by the amount of literature that was accessible on the topic; however, the global approach seemed disjointed due to the challenge of availing policies and guidelines that can equitably address the various socioeconomic and regional situations. The available literature was also not categorised or aligned within specific themes.

As specified in Article 4, paragraph 3 of the Minamata Convention on Mercury, parties were mandated to develop national phase-down guidelines, and these were at an advanced stage in regions such as Europe. The majority of the articles reviewed implied a phase-down strategy to be implemented between 2020 and 2030; we are presently in this crucial timeframe hence documentation of implementation efforts should be clearly presented for ease of reference. It was also clear that countries are at different levels of adopting the dental amalgam phase-down. While some HIC stopped the usage of amalgam more than 15 years ago, others particularly in LMIC continued to report high usage levels characterised by funding and policy limitations which impacted negatively on awareness of the phase-down and competencies required for alternatives. Moreover, in both HIC and LMIC, concerns remained regarding cost implications of alternatives, especially for underprivileged populations. Therefore, stakeholder engagement remains an important factor if the dental amalgam phase-down is to be achieved globally.

A lot of the literature reviewed here was low-level evidence cross-sectional surveys, followed by a few review articles, meeting proceedings and opinion articles. This is an evident gap and more high-level evidence studies are necessary to aid in decision making, particularly regarding dental amalgam alternatives and requisite clinician skills and competencies. While the scope of this review did not include a comparison of the performance of alternative restorative dental materials largely because the available studies were not conducted specifically within the context of the phase-down, they were highlighted as a baseline from which new systematic reviews may be conducted to address this gap.

The perceptions on these alternatives showed a mixed picture of clinician's experiences regarding selection, skill, cost and patient preference in both HIC and LMIC. Nonetheless, it was encouraging to note that more time was being allocated towards the teaching and placement of posterior composites and other DAARs in dental schools globally, and often among younger clinicians, composite restorations were preferred to amalgam. It is noteworthy that there is no single restorative dental biomaterial that can meet all the needs of the patient and the clinician so as the search for viable alternatives continues, it is inspiring that the available ones are promising.

Finally, it was also motivating that globally, there was evidence of phase-down strategies through the development of dental school curricula that demonstrated a paradigm shift from the amalgam era and G.V. Black's principles of 'extension for prevention' to modern teaching in preventive, minimal intervention and adhesive dentistry. The usage of adhesive, aesthetic materials had increased, particularly among younger dentists, who were also more confident in the handling of these materials. Although disparities in oral disease burden, oral health training, oral healthcare provision and funding are important considerations, there is a need for synergized efforts among all stakeholders for inclusive policies which consider the regional variations yet enable adoption of a global phase-down strategy. It cannot be overemphasised that the best strategy for the dental amalgam phase-down should begin with oral health promotion and caries prevention so as to minimise the need for restorations.

## Figures and Tables

**Table 1:** Summary of reviewed literature.

Author (year of study)	Country of study	Study design	Participants	Emergent themes	Reference
Skjelvik and Grytli (2012)	Norway	Cross-sectional survey	Dentists	Global status of the dental amalgam phase-down	[24]
Alexander et al. (2014)	—	Review	—		[26]
Lynch and Wilson (2013)	—	Opinion article	—		[7]
Alexander et al. (2014)	Australia	Questionnaire survey	Dentists		[27]
Alexander et al. (2017)	Australia	Qualitative study	Dentists		[28]
Lombard et al. (2009)	South Africa	Questionnaire survey	Heads of restorative departments		[29]
Lynch and Wilson (2010)	United Kingdom (UK) and Ireland	Questionnaire survey	Heads of dental schools		[30]
Castillo-de Oyagüe et al. (2012)	Spain	Questionnaire survey	Heads of restorative departments		[31]
Fukushima et al. (2000)	Japan	Questionnaire survey	Heads of restorative departments		[32]
Forss and Widström (2001)	Finland	Questionnaire survey	Dentists		[33]
Zoebali et al. (2022)	Kenya	Questionnaire survey	Final year dental students and dental interns		[34]
Lynch et al. (2010)	UK and Ireland	Questionnaire survey	Heads of dental schools		[35]
Ben-Gal and Weiss (2011)	Israel	Questionnaire survey	Heads of dental schools		[36]
O'Sullivan et al. (2012)	Ireland	Questionnaire survey	Heads of dental schools		[37]
Lynch et al. (2007)	USA, Canada, Ireland and UK	Comparative review	—		[38]
Alreshaid et al. (2021)	Canada	Questionnaire survey/data entry	Chairs/heads of restorative departments and clinic directors of dental schools		[39]
Alreshaid et al. (2023)	USA	Questionnaire survey/data entry	Chairs/heads of restorative departments and clinic directors of dental schools		[40]
Broadbent et al. (2020)	New Zealand	Cross-sectional study	Patient records		[41]
Alkhudhairy (2016)	Saudi Arabia	Cross-sectional questionnaire survey	Dentists and dental interns		[42]

Skjelvik and Grytli (2012)	Norway	Cross-sectional survey	Dentists	Status of the dental amalgam phase-down in HIC	[24]
Edlich et al. (2008)	—	Opinion article	—		[43]
Petersen et al. (2010)	—	Meeting proceedings	WHO oral health representatives		[44]
Kopperud et al. (2016)	Norway	Questionnaire survey	Dentists		[45]
Forss and Widström (2001)	Finland	Questionnaire survey	Dentists		[33]
Edlich et al. (2008)	—	Opinion article	—		[46]
Loch et al. (2019)	Oceania	Questionnaire survey	Heads of dental schools		[47]
Alreshaid et al. (2021)	Canada	Questionnaire survey/data entry	Chairs/heads of restorative departments and clinic directors of dental schools		[39]
Alreshaid et al. (2023)	USA	Questionnaire survey/data entry	Chairs/heads of restorative departments and clinic directors of dental schools		[40]
Broadbent et al. (2020)	New Zealand	Cross-sectional study	Patient records		[41]
Alkhudhairy (2016)	Saudi Arabia	Cross-sectional questionnaire survey	Dentists and dental interns		[42]
Bakhurji et al. (2017)	USA	Questionnaire survey	General and paediatric dentists		[13]
Aggarwal et al. (2019)	UK	Mixed-methods	NHS data sets, dentists, dental school teaching leads and NHS dental commissioners		[48]
Mikulás et al. (2018)	—	Review	—		[49]

Osiro et al. (2019)	—	Opinion article	—	Status of the dental amalgam phase-down in LMIC	[50]
Faraj et al. (2015)	Iraq	Cross-sectional questionnaire survey	Dentists		[51]
Udoye and Aguwa (2008)	Nigeria	Questionnaire survey	Dentists		[52]
Kisumbi et al. (2013)	East Africa (Kenya, Tanzania, Uganda)	Questionnaire survey	Dentists		[53]
Ganatra et al. (2009)	Kenya	Survey	Dentists		[54]
Osiro et al. (2016)	Kenya	Questionnaire survey	Dentists		[55]
Umesi et al. (2020)	Nigeria	Cross-sectional study	Patient records		[56]
Lombard et al. (2009)	South Africa	Questionnaire survey	Heads of restorative departments		[29]
Zoebali et al. (2022)	Kenya	Questionnaire survey	Final year dental students and dental interns		[34]
Ramesh et al. (2019)	India	Questionnaire survey	Dental clinics		[57]
Al-Asmar et al. (2019)	Jordan	Cross-sectional questionnaire survey	Dentists		[58]
Khan et al. (2022)	Pakistan	Cross-sectional questionnaire survey	Dentists		[59]
Arotiba et al. (2019)	Nigeria	Meeting proceedings	Dentists		[60]
Arotiba et al. (2020)	Nigeria	Review	—		[61]
Makanjuola et al. (2020)	Nigeria	Cross-sectional questionnaire survey	Dentists and dental students		[62]
Chala et al. (2012)	Burkina Faso and Morocco	Cross-sectional questionnaire survey	Dentists		[63]
Osamong et al. (2005)	Kenya	Cross-sectional questionnaire survey	Dentists		[64]
Sawair et al. (2010)	Jordan	Cross-sectional questionnaire survey	Dentists		[65]
Kefi et al. (2011)	Pakistan	Cross-sectional questionnaire survey	Dentists		[66]
Chaari et al. (2009)	Tunisia	Questionnaire survey	Dentists and dental assistants		[67]
Sarr et al. (2005)	Senegal	Questionnaire survey	Dentists		[68]
Lollobrigida de Souza et al. (2012)	Brazil	Survey and lab assays	Public and private dental clinics		[69]

Bailey et al. (2022)	UK	Questionnaire survey	Dentists	Perceptions on DAAR materials	[70]
Bailey et al. (2022)	UK	Questionnaire survey	Dentists		[71]
Makanjuola et al. (2020)	Nigeria	Cross-sectional questionnaire survey	Dentists and dental students		[62]
Kopperud et al. (2016)	Norway	Questionnaire survey	Dentists		[45]
Nascimento et al. (2010)	USA and Scandinavia	Questionnaire survey	Dentists		[72]
Umesi et al. (2020)	Nigeria	Cross-sectional study	Patient records		[56]
Alexander et al. (2016)	Australia	Questionnaire survey	Dentists		[73]
Alexander et al. (2017)	Australia	Questionnaire survey	Dentists		[28]
Bayne et al. (2013)	—	Opinion article	—		[74]
Worthington et al. (2021)	—	Systematic review	—		[75]
Alcaraz et al. (2014)	—	Systematic review	—		[15]
Kielbassa et al. (2016)	—	Systematic review	—		[76]
Kielbassa et al. (2017)	—	Systematic review	—		[77]
Aggarwal et al. (2019)	UK	Mixed-methods	NHS data sets, dentists, dental school teaching leads and NHS dental commissioners		[48]
Alexander et al. (2014)	Australia	Questionnaire survey	Dentists		[27]
Austin et al. (2016)	UK	Meeting proceedings	Prosthodontists		[78]
Schmalz et al. (2024)	—	Concise review	—		[79]
Bailey et al. (2022)	UK	Discrete choice experiment	Dentists		[80]
Al-Asmar et al. (2019)	Jordan	Cross-sectional questionnaire survey	Dentists		[58]

Bakhurji et al. (2019)	USA	Choice-based conjoint analysis	Paediatric dentists	Dental amalgam phase-down approaches	[81]
Kielbassa et al. (2016)	—	Systematic review	—		[76]
Kielbassa et al. (2017)	—	Systematic review	—		[77]
Mackey et al. (2014)	—	Opinion article	—		[82]

Al-Rabab'ah et al. (2016)	Jordan	Questionnaire survey	Dentists	Dentists' knowledge, skill, competency and attitude	[83]
Zoebali et al. (2022)	Kenya	Questionnaire survey	Final year dental students and dental interns		[34]
Kopperud et al. (2016)	Norway	Questionnaire survey	Dentists		[45]
Alexander et al. (2017)	Australia	Questionnaire survey	Dentists		[28]
Lynch and Wilson (2013)	—	Opinion article	—		[23]
Alexander et al. (2016)	Australia	Questionnaire survey	Dentists		[73]
Callanan et al. (2020)	Ireland	Cross-sectional survey	Dentists		[84]
Alexander et al. (2020)	Australia	Mixed-methods	Dental educators		[85]

Lombard et al. (2009)	South Africa	Questionnaire survey	Heads of restorative departments	Dental amalgam phase-down curricula and policy	[29]
Lynch et al. (2010)	UK and Ireland	Questionnaire survey	Heads of dental schools		[30]
Fukushima et al. (2000)	Japan	Questionnaire survey	Heads of restorative departments		[32]
Ben-Gal and Weiss (2011)	Israel	Cross-sectional study	Patient records		[36]
Sadeghi et al. (2009)	Iran	Questionnaire survey	Heads of operative dentistry at dental schools		[86]
Schulte et al. (2011)	24 European and three North/South American countries	Meeting proceedings	Members of ORCA and ADEE		[87]
Martignon et al. (2014)	Colombia	Meeting proceedings	Representatives from dental schools		[88]
Martignon et al. (2013)	Latin America	Questionnaire survey	Cariology instructors at Dental schools		[89]
Fontana et al. (2016)	USA and Canada	Meeting proceedings	Representatives of dental schools		[90]
Loch et al. (2019)	Oceania	Questionnaire survey	Heads of dental schools		[47]
Bailey et al. (2022)	UK	Questionnaire survey	Dentists		[70]
Makanjuola et al. (2020)	Nigeria	Cross-sectional questionnaire survey	Dentists and dental students		[62]
Umesi et al. (2020)	Nigeria	Cross-sectional study	Patient records		[56]
Alexander et al. (2020)	Australia	Mixed-methods	Dental educators		[85]
Al-Rabab'ah et al. (2016)	Jordan	Questionnaire survey	Dentists		[83]
Lynch and Wilson (2013)	—	Opinion article	—		[23]
Sidhu et al. (2021)	Malaysia	Cross-sectional questionnaire survey	Dental school faculty members		[91]
Alexander et al. (2017)	Australia	Questionnaire survey	Dentists		[28]
Broadbent et al. (2020)	New Zealand	Cross-sectional study	Patient records		[41]
Alreshaid et al. (2021)	Canada	Questionnaire survey/data entry	Chairs/heads of restorative departments and clinic directors of dental schools		[39]
Alreshaid et al. (2023)	USA	Questionnaire survey/data entry	Chairs/heads of restorative departments and clinic directors of dental schools		[40]

## Data Availability

The data that support the findings of this study are available from the corresponding author upon reasonable request.

## References

[1] Hachiya N. (2006). The History and the Present of Minamata Disease. *Japan Medical Association Journal*.

[2] Buttke D. E. (2011). Toxicology, Environmental Health, and the “One Health” Concept. *Journal of Medical Toxicology*.

[3] (UNEP) UNEP. Minamata Convention on Mercury (2013). United Nations Environment Programme.

[4] Anusavice K. J., Shen C., Rawls H. R. (2012). *Phillips’ Science of Dental Materials*.

[5] Futsaeter G., Wilson S. The UNEP Global Mercury Assessment: Sources, Emissions and Transport.

[6] Fisher J., Varenne B., Narvaez D., Vickers C. (2018). The Minamata Convention and the Phase Down of Dental Amalgam. *Bulletin of the World Health Organization*.

[7] Lynch C. D., Wilson N. H. F. (2013). Managing the Phase-Down of Amalgam: Part II. Implications for Practising Arrangements and Lessons From Norway. *British Dental Journal*.

[8] Khairiyah A. M., Razak I. A., Raja-Latifah R. J. (2009). Costing Dental Restorations in Public Sector Dental Clinics. *Asia Pacific Journal of Public Health*.

[9] Sjögren P. (2006). Cost of Composite and Glass Ionomer Class II Molar Restorations and Theoretical Analyses of Cost per Year of Function at Public Dental Services in Sweden. *Swedish dental journal*.

[10] Bailey O. (2025). The Long-Term Oral Health Consequences of an Amalgam Phase-Out. *British Dental Journal*.

[11] Mitchell R. J., Koike M., Okabe T. (2007). Posterior Amalgam Restorations—Usage, Regulation, and Longevity. *Dental Clinics of North America*.

[12] Kelly P. G., Smales R. J. (2004). Long-Term Cost-Effectiveness of Single Indirect Restorations in Selected Dental Practices. *British Dental Journal*.

[13] Bakhurji E., Scott T., Mangione T., Sohn W. (2017). Dentists’ Perspective About Dental Amalgam: Current Use and Future Direction. *Journal of Public Health Dentistry*.

[14] Diamantopoulou E. I., Plastiras O. E., Mourouzis P., Samanidou V. (2020). Validation of a Simple HPLC–UV Method for the Determination of Monomers Released From Dental Resin Composites in Artificial Saliva. *Methods and Protocols*.

[15] Alcaraz M. G. R., Veitz-Keenan A., Sahrmann P., Schmidlin P. R., Davis D., Iheozor-Ejiofor Z. (2014). Direct Composite Resin Fillings Versus Amalgam Fillings for Permanent or Adult Posterior Teeth. *Cochrane Database of Systematic Reviews*.

[16] Iano F. G., Dos Santos Sobrinho O., Da Silva T. L. (2008). Optimizing the Procedure for Mercury Recovery From Dental Amalgam. *Brazilian Oral Research*.

[17] George G. N., Singh S. P., Hoover J., Pickering I. J. (2009). The Chemical Forms of Mercury in Aged and Fresh Dental Amalgam Surfaces. *Chemical Research in Toxicology*.

[18] Eley B. M. (1997). The Future of Dental Amalgam: A Review of the Literature. Part 6: Possible Harmful Effects of Mercury From Dental Amalgam. *British Dental Journal*.

[19] Bates M. N. (2006). Mercury Amalgam Dental Fillings: An Epidemiologic Assessment. *International Journal of Hygiene and Environmental Health*.

[20] Ericson D., Kidd E., McComb D., Mjör I., Noack M. J. (2003). Minimally Invasive Dentistry--Concepts and Techniques in Cariology. *Oral Health & Preventive Dentistry*.

[21] Pitts N. (2011). Preventive and Minimal Intervention Dentistry in the Undergraduate Curriculum. *Journal of Dentistry*.

[22] Shoaee S., Ghasemian A., Mehrabani K. (2015). Burden of Oral Diseases in Iran, 1990-2010: Findings From the Global Burden of Disease Study 2010. *Archives of Iranian medicine*.

[23] Lynch C. D., Wilson N. H. F. (2013). Managing the Phase-Down of Amalgam: Part I. Educational and Training Issues. *British Dental Journal*.

[24] Skjelvik J. M., Grytli E. S. (2012). Review of Norwegian Experiences With the Phase-Out of Dental Amalgam Use. *Oslo: Norwegian Climate and Pollution Agency*.

[25] Minamata Convention on Mercury (2025). https://minamataconvention.org/en/parties/overview.

[26] Alexander G., Hopcraft M. S., Tyas M. J., Wong R. H. K. (2014). Dentists’ Restorative Decision-Making and Implications for an ‘Amalgamless’ Profession. Part 1: A Review. *Australian Dental Journal*.

[27] Alexander G., Hopcraft M. S., Tyas M. J., Wong R. H. K. (2014). Dentists’ Restorative Decision-Making and Implications for an ‘Amalgamless’ Profession. Part 2: A Qualitative Study. *Australian Dental Journal*.

[28] Alexander G., Hopcraft M. S., Tyas M. J., Wong R. H. K. (2017). Dentists’ Restorative Decision-Making and Implications for an ‘Amalgamless’ Profession. Part 4: Clinical Factor. *Australian Dental Journal*.

[29] Lombard R., Du Preez I. C., Oberholzer T. G., Gugushe T. S. (2009). Teaching Approaches in South African Dental Schools: Direct Restorative Procedures. *South African Dental Journal*.

[30] Lynch C. D., Wilson N. H. F. (2010). Teaching of Direct Posterior Resin Composite Restorations in UK Dental Therapy Training Programmes. *British Dental Journal*.

[31] Castillo-De Oyague R., Lynch C., McConnell R., Wilson N. (2012). Teaching the Placement of Posterior Resin-Based Composite Restorations in Spanish Dental Schools. *Medicina Oral Patología Oral y Cirugia Bucal*.

[32] Fukushima M., Iwaku M., Setcos J. C., Wilson N. H. F., Mjör I. A. (2000). Teaching of Posterior Composite Restorations in Japanese Dental Schools. *International Dental Journal*.

[33] Forss H., Widström E. (2009). From Amalgam to Composite: Selection of Restorative Materials and Restoration Longevity in Finland. *Acta Odontologica Scandinavica*.

[34] Zoebali M., Osiro O. A., James R. M. (2022). Minamata Convention on Mercury: An Online Survey on Knowledge and Attitude Towards Restorative Practice Among Dental Students and Dental Interns in Kenya. *East African Medical Journal*.

[35] Lynch C. D., Frazier K. B., McConnell R. J., Blum I. R., Wilson N. H. F. (2010). State-of-the-Art Techniques in Operative Dentistry: Contemporary Teaching of Posterior Composites in UK and Irish Dental Schools. *British Dental Journal*.

[36] Ben-Gal G., Weiss E. I. (2011). Trends in Material Choice for Posterior Restorations in an Israeli Dental School: Composite Resin Versus Amalgam. *Journal of Dental Education*.

[37] O’Sullivan C. O., McKenna G. J., Burke F. M. (2012). Trends in Material Choice for Direct Restorations by Final Year Students From University College Cork 2004-2009. *The European Journal of Prosthodontics and Restorative Dentistry*.

[38] Lynch C. D., McConnell R. J., Wilson N. H. F. (2007). Trends in the Placement of Posterior Composites in Dental Schools. *Journal of Dental Education*.

[39] Alreshaid L., El-Badrawy W., Lawrence H. P., Santos M. J., Prakki A. (2021). Composite Versus Amalgam Restorations Placed in Canadian Dental Schools. *Operative Dentistry*.

[40] Alreshaid L., El-Badrawy W., Kulkarni G., Santos M. J., Prakki A. (2023). Resin Composite Versus Amalgam Restorations Placed in United States Dental Schools. *Operative Dentistry*.

[41] Broadbent J. M., Murray C. M., Schwass D. R. (2020). The Dental Amalgam Phasedown in New Zealand: A 20-Year Trend. *Operative Dentistry*.

[42] Alkhudhairy F. (2016). Attitudes of Dentists and Interns in Riyadh to the Use of Dental Amalgam. *BMC Research Notes*.

[43] Edlich R., Cross C. L., Dahlstrom J. J., Long III W. B., Newkirk A. T. (2008). Implementation of Revolutionary Legislation for Informed Consent for Dental Patients Receiving Amalgam Restorations. *Journal of Environmental Pathology, Toxicology and Oncology*.

[44] Petersen PE, Baez R, Kwan S, Ogawa H, World Health Organization Future Use of Materials for Dental Restoration: Report of the Meeting Convened at WHO HQ, Geneva, Switzerland 16^th^ to 17^th^ November 2009.

[45] Kopperud S. E., Staxrud F., Espelid I., Tveit A. B. (2016). The Post-Amalgam Era: Norwegian Dentists’ Experiences With Composite Resins and Repair of Defective Amalgam Restorations. *International Journal of Environmental Research and Public Health*.

[46] Edlich R. F., Cochran A. A., Cross C. L., Wack C. A., Long W. B., Newkirk A. T. (2008). Legislation and Informed Consent Brochures for Dental Patients Receiving Amalgam Restorations. *International Journal of Toxicology*.

[47] Loch C., Liaw Y., Metussin A. P. (2019). The Teaching of Posterior Composites: A Survey of Dental Schools in Oceania. *Journal of Dentistry*.

[48] Aggarwal V. R., Pavitt S., Wu J. (2019). Assessing the Perceived Impact of Post Minamata Amalgam Phase Down on Oral Health Inequalities: A Mixed-Methods Investigation. *BMC Health Services Research*.

[49] Mikulás K., Linninger M., Takács E. (2018). Paradigm Shift in Conservative Dentistry: The End of the Amalgam Era. *Orvosi hetilap*.

[50] Osiro O. A., Kariuki D. K., Gathece L. W. (2019). The Minamata Convention on Mercury and Its Implications for Management of Dental Caries in Low- and Middle-Income Countries. *International Dental Journal*.

[51] Faraj B. M., Mohammad H. M., Mohammad K. M. (2015). The Changes in Dentists’ Perception and Patient’s Acceptance on Amalgam Restoration in Kurdistan-Iraq: A Questionnaire-Based Cross-Sectional Study. *Journal of Clinical and Diagnostic Research*.

[52] Udoye C., Aguwa E. (2008). Amalgam Safety and Dentists’ Attitude: A Survey Among a Subpopulation of Nigerian Dentists. *Operative Dentistry*.

[53] Kisumbi B. K., Gathece L. W., Koyio L. N., Wamai J. (2013). Dental Amalgam Waste Management by Dentists in East Africa. *International Dental Journal*.

[54] Ganatra F. A., Kisumbi B. K., Gathece L. W. (2009). Selection of Posterior Dental Restoratives by Dentists. *Journal of Kenya Dental Association*.

[55] Osiro O. A., Kisumbi B. K., Simila H. O. (2016). Selection of Direct Restorative and Root Filling Materials Kenyan Dentists in 2016. *East Africa Medical Journal*.

[56] Umesi D. C., Oremosu O. A., Makanjuola J. O. (2020). Amalgam Phase Down: Baseline Data Preceding Implementation in Nigeria. *International Dental Journal*.

[57] Ramesh K. K., Ramesh M., Krishnan R. (2019). Management and Disposal of Mercury and Amalgam in the Dental Clinics of South India: A Cross-Sectional Study. *Journal of Pharmacy And Bioallied Sciences*.

[58] Al-Asmar A. A., Al-Khatib K. M., Al-Amad T. Z., Sawair F. A. (2019). Has the Implementation of the Minamata Convention Had an Impact on the Practice of Operative Dentistry in Jordan?. *Journal of International Medical Research*.

[59] Khan S., Khalid N., Bajwa O., Qamar T., Kazmi A., Tariq A. (2022). Amalgam Phase-Out, an Environmental Safety Concern: A Cross-Sectional Study Among General Dental Practitioners in Pakistan. *Eastern Mediterranean Health Journal*.

[60] Arotiba G. T., Loto A. O., Ijarogbe O. (2019). Lessons From Mercury Dental Amalgam Phase Down for Developing Economies. *African Journal of Oral Health*.

[61] Arotiba G. T., Ijarogbe O. A., Awotile A. O. (2020). Accelerating the Phase Down of Dental Amalgam in Africa and Developing Economies: A Leapfrogging Strategy. *Oral Health Dental Science*.

[62] Makanjuola J. O., Umesi D. C., Ndukwe A. N. (2020). Managing the Phase-Down of Amalgam Amongst Nigerian Dental Professionals and Students: A National Survey. *European Journal of Dental Education*.

[63] Chala S., Sawadogo A., Sakout M., Abdallaoui F. (2012). Management of Wastes From Dental Amalgam by Dentists in Burkina Faso and Morocco. *Odonto-Stomatologie Tropicale = Tropical Dental Journal*.

[64] Osamong L. A., Gathece L. W., Kisumbi B. K., Mutave R. J. (2005). Management of Dental Waste by Practitioners in Nairobi, Kenya. *African Journal of Oral Health*.

[65] Sawair F., Hassoneh Y., Jamleh A., Al-Rabab’ah M. (2010). Observance of Proper Mercury Hygiene Practices by Jordanian General Dental Practitioners. *International Journal of Occupational Medicine and Environmental Health*.

[66] Kefi K. I., Maria M. A., Majid M. Z. (2011). Dental Amalgam: Effects of Alloy/Mercury Mixing Ratio, Uses and Waste Management. *Journal of Ayub Medical College Abbottabad*.

[67] Chaari N., Kerkeni A., Saadeddine S., Neffati F., Khalfallah T., Akrout M. (2009). Mercury Impregnation in Dentists and Dental Assistants in Monastir City, Tunisia. *Revue de Stomatologie et de Chirurgie Maxillo-faciale*.

[68] Sarr M., Kane A. W., Toure B., Faye B., Faye D., Ndoye N. N. (2005). Risk Behavior Associated With the Manipulation of Dental Amalgam in Senegal. *Odonto-Stomatologie Tropicale = Tropical Dental Journal*.

[69] Lollobrigida De Souza J. P. B., Nozawa S. R., Honda R. T. (2012). Improper Waste Disposal of Silver-Mercury Amalgam. *Bulletin of Environmental Contamination and Toxicology*.

[70] Bailey O., Vernazza C. R., Stone S., Ternent L., Roche A.-G., Lynch C. (2022). Amalgam Phase-Down Part 2: UK-Based Knowledge, Opinions, and Confidence in the Alternatives. *JDR Clinical & Translational Research*.

[71] Bailey O., Vernazza C. R., Stone S., Ternent L., Roche A.-G., Lynch C. (2022). Amalgam Phase-Down Part 1: UK-Based Posterior Restorative Material and Technique Use. *JDR Clinical & Translational Research*.

[72] Nascimento M. M., Gordan V. V., Qvist V. (2010). Reasons for Placement of Restorations on Previously Unrestored Tooth Surfaces by Dentists in The Dental Practice-Based Research Network. *The Journal of the American Dental Association*.

[73] Alexander G., Hopcraft M. S., Tyas M. J., Wong R. H. K. (2016). Dentists’ Restorative Decision-Making and Implications for an ‘Amalgamless’ Profession. Part 3: Dentists’ Attitudes. *Australian Dental Journal*.

[74] Bayne S., Petersen P. E., Piper D., Schmalz G., Meyer D. (2013). The Challenge for Innovation in Direct Restorative Materials. *Advances in Dental Research*.

[75] Worthington H.V., Khangura S., Seal K. (2021). Direct Composite Resin Fillings Versus Amalgam Fillings for Permanent Posterior Teeth. *The Cochrane Database of Systematic Reviews*.

[76] Kielbassa A. M., Glockner G., Wolgin M., Glockner K. (2016). Systematic Review on Highly Viscous Glass-Ionomer Cement/Resin Coating Restorations (Part I): Do They Merge Minamata Convention and Minimum Intervention Dentistry?. *Quintessence International*.

[77] Kielbassa A. M., Glockner G., Wolgin M., Glockner K. (2017). Systematic Review on Highly Viscous Glass-Ionomer Cement/Resin Coating Restorations (Part II): Do They Merge Minamata Convention and Minimum Intervention Dentistry?. *Quintessence International*.

[78] Austin R., Eliyas S., Burke F. J. T., Taylor P., Toner J., Briggs P. (2016). British Society of Prosthodontics Debate on the Implications of the Minamata Convention on Mercury to Dental Amalgam—Should Our Patients be Worried?. *Dental Update*.

[79] Schmalz G., Schwendicke F., Hickel R., Platt J. A. (2024). Alternative Direct Restorative Materials for Dental Amalgam: A Concise Review Based on an FDI Policy Statement. *International Dental Journal*.

[80] Bailey O., Stone S., Ternent L., Vernazza C. R. (2022). Public Valuation of Direct Restorations: A Discrete Choice Experiment. *Journal of Dental Research*.

[81] Bakhurji E., Scott T., Sohn W. (2019). Factors Associated With Pediatric Dentists’ Choice of Amalgam: Choice-Based Conjoint Analysis Approach. *JDR Clinical & Translational Research*.

[82] Mackey T. K., Contreras J. T., Liang B. A. (2014). The Minamata Convention on Mercury: Attempting to Address the Global Controversy of Dental Amalgam Use and Mercury Waste Disposal. *Science of The Total Environment*.

[83] Al-Rabab’ah M. A., Bustani M. A., Khraisat A. S., Sawair F. A. (2016). Phase Down of Amalgam: Awareness of Minamata Convention Among Jordanian Dentists. *Saudi Medical Journal*.

[84] Callanan A., Harding M., Lynch C. D., Burke F. M., Hayes M. (2020). Dentists’ Attitudes Towards the Phase-Down of Dental Amalgam in Ireland. *Journal of the Irish Dental Association*.

[85] Alexander G., Hopcraft M. S., Tyas M. J., Wong R. H. K. (2020). Dental Educators’ Attitudes Towards the Teaching of Dental Amalgam. *European Journal of Dental Education*.

[86] Sadeghi M., Lynch C. D., Wilson N. H. F. (2009). Trends in Dental Education in the Persian Gulf--An Example From Iran: Contemporary Placement of Posterior Composites.. *The European Journal of Prosthodontics and Restorative Dentistry*.

[87] Schulte A. G., Pitts N. B., Huysmans M., Splieth C., Buchalla W. (2011). European Core Curriculum in Cariology for Undergraduate Dental Students. *European Journal of Dental Education*.

[88] Martignon S., Marín L. M., Pitts N., Jácome-Liévano S. (2014). Consensus on Domains, Formation Objectives and Contents in Cariology for Undergraduate Dental Students in Colombia. *European Journal of Dental Education*.

[89] Martignon S., Gomez J., Tellez M., Ruiz J. A., Marin L. M., Rangel M. C. (2013). Current Cariology Education in Dental Schools in Spanish–Speaking Latin American Countries. *Journal of Dental Education*.

[90] Fontana M., Guzmán-Armstrong S., Schenkel A. B. (2016). Development of a Core Curriculum Framework in Cariology for U.S. Dental Schools. *Journal of Dental Education*.

[91] Sidhu P., Sultan O. S., Math S. Y. (2021). Current and Future Trends in the Teaching of Direct Posterior Resin Composites in Malaysian Dental Schools: A Cross-Sectional Study. *Journal of Dentistry*.

[92] Kassebaum N. J., Bernabé E., Dahiya M., Bhandari B., Murray C. J. L., Marcenes W. (2015). Global Burden of Untreated Caries. *Journal of Dental Research*.

[93] Mukashyaka C., Uzabakiriho B., Amoroso C. L. (2015). Dental Caries Management at a Rural District Hospital in Northern Rwanda: A Neglected Disease. *Public Health Action*.

[94] United Nations Environment Programme (2025). Lessons From Countries Phasing Down Dental Amalgam Use 2016. https://www.unep.org/globalmercurypartnership/resources/report/.

[95] Government U. K. (2025). National Plan to Phase Down Use of Dental Amalgam in England 2019. https://www.gov.uk/government/publications/.

[96] European Union (2025). Regulation (EU) 2024/1849 of the European Parliament and of the Council of 13 June 2024 Amending Regulation (EU) 2017/852 on Mercury as Regards Dental Amalgam and Other Mercury-Added Products Subject to Export, Import and Manufacturing Restrictions 2024.

[97] Peres M. A., Macpherson L. M. D., Weyant R. J. (2019). Oral Diseases: A Global Public Health Challenge. *The Lancet*.

[98] Fugolin A. P. P., Pfeifer C. S. (2017). New Resins for Dental Composites. *Journal of Dental Research*.

[99] Gupta N., Jaiswal S., Nikhil V., Gupta S., Jha P., Bansal P. (2019). Comparison of Fluoride Ion Release and Alkalizing Potential of a New Bulk-Fill Alkasite. *Journal of Conservative Dentistry*.

[100] Ilie N., Bucuta S., Draenert M. (2013). Bulk-Fill Resin-Based Composites: An In Vitro Assessment of Their Mechanical Performance. *Operative Dentistry*.

[101] Nascimento A. S., Rodrigues J. F. B., Torres R. H. N. (2019). Physicomechanical and Thermal Analysis of Bulk-Fill and Conventional Composites. *Brazilian Oral Research*.

[102] Petersen R. C., Reddy M. S., Liu P.-R. (2018). Fiber-Reinforced Composites: A Breakthrough in Practical Clinical Applications With Advanced Wear Resistance for Dental Materials.. *EC Dental Science*.

[103] Klinke T., Daboul A., Turek A., Frankenberger R., Hickel R., Biffar R. (2016). Clinical Performance During 48 Months of Two Current Glass Ionomer Restorative Systems With Coatings: A Randomized Clinical Trial in the Field. *Trials*.

[104] Hopp C. D., Land M. F. (2013). Considerations for Ceramic Inlays in Posterior Teeth: A Review. *Clinical, Cosmetic and Investigational Dentistry*.

[105] Balkaya H., Arslan S., Pala K. (2019). A Randomized, Prospective Clinical Study Evaluating Effectiveness of a Bulk-Fill Composite Resin, a Conventional Composite Resin and a Reinforced Glass Ionomer in Class II Cavities: One-Year Results. *Journal of Applied Oral Science*.

[106] Van Dijken J. W. V., Lindberg A. (2015). A 15-Year Randomized Controlled Study of a Reduced Shrinkage Stress Resin Composite. *Dental Materials*.

[107] Poon E. C. M., Smales R. J., Yip K. H.-K. (2005). Clinical Evaluation of Packable and Conventional Hybrid Posterior Resin-Based Composites: Results at 3.5 Years. *The Journal of the American Dental Association*.

[108] NMRde A., FBde S., RVf D., PKbdaS L., AUd B., Montenegro R. V. (2022). Longevity of Bulk Fill and Ormocer Composites in Permanent Posterior Teeth: Systematic Review and Meta-Analysis. *American Journal of Dentistry*.

[109] Da Veiga AMA., Cunha A. C., Ferreira DMTP. (2016). Longevity of Direct and Indirect Resin Composite Restorations in Permanent Posterior Teeth: A Systematic Review and Meta-Analysis. *Journal of Dentistry*.

[110] Ngo H., Opsahl-Vital S. (2014). Minimal Intervention Dentistry II: Part 7. Minimal Intervention in Cariology: The Role of Glass-Ionomer Cements in the Preservation of Tooth Structures Against Caries. *British Dental Journal*.

[111] Powers J. M., Burgess J. O. Performance Standards for Competitive Dental Restorative Materials.

[112] Chalissery V. P., Marwah N., Almuhaiza M. (2016). Study of the Mechanical Properties of the Novel Zirconia-Reinforced Glass Ionomer Cement. *The Journal of Contemporary Dental Practice*.

[113] Antonucci J. M. (1988). Resin-Modified Glass Ionomer Cement. *U.S. Patent Application*.

[114] Sidhu S. K., Watson T. F. (1995). Resin-Modified Glass Ionomer Materials. A Status Report for the American Journal of Dentistry. *American Journal of Dentistry*.

[115] Maldonado A., Swartz M. L., Phillips R. W. (1978). An In Vitro Study of Certain Properties of a Glass Ionomer Cement. *The Journal of The American Dental Association*.

[116] Baroudi K., Ibraheem S. N. (2015). Assessment of Chair-Side Computer-Aided Design and Computer-Aided Manufacturing Restorations: A Review of the Literature.. *Journal of International Oral Health: JIOH*.

[117] Mörmann W. H., Brandestini M., Lutz F. (1987). The Cerec System: Computer-Assisted Preparation of Direct Ceramic Inlays in 1 Setting. *Quintessenz*.

[118] Rosenstiel S. F., Land M. F., Rashid R. G. (2004). Dentists’ Molar Restoration Choices and Longevity: A Web-Based Survey. *The Journal of Prosthetic Dentistry*.

[119] Mulic A., Svendsen G., Kopperud S. E. (2018). A Retrospective Clinical Study on the Longevity of Posterior Class II Cast Gold Inlays/Onlays. *Journal of Dentistry*.

[120] Bandlish L. K., Mariatos G. (2009). Long-Term Survivals of ’Direct-Wax’ Cast Gold Onlays: A Retrospective Study in a General Dental Practice. *British Dental Journal*.

[121] Small B. (2000). The Use of Cast Gold Restorations: Scientific Basis and Clinical Technique. *Dentistry Today*.

[122] Innes N. P. T., Ricketts D., Chong L. Y., Keightley A. J., Lamont T., Santamaria R. M. (2015). Preformed Crowns for Decayed Primary Molar Teeth. *Cochrane Database of Systematic Reviews*.

[123] Seale N. S., Randall R. (2015). The Use of Stainless Steel Crowns: A Systematic Literature Review. *Pediatric Dentistry*.

[124] Mathew M. G., Roopa K. B., Soni A. J., Khan M. M., Kauser A. (2020). Evaluation of Clinical Success, Parental and Child Satisfaction of Stainless Steel Crowns and Zirconia Crowns in Primary Molars. *Journal of Family Medicine and Primary Care*.

[125] Zero D., Fontana M., Lennon Á. M. (2001). Clinical Applications and Outcomes of Using Indicators of Risk in Caries Management. *Journal of Dental Education*.

[126] Lynch C. D., Frazier K. B., McConnell R. J., Blum I. R., Wilson N. H. F. (2011). Minimally Invasive Management of Dental Caries: Contemporary Teaching of Posterior Resin-Based Composite Placement in US and Canadian Dental Schools. *The Journal of the American Dental Association*.

[127] Holmgren C. J., Roux D., Doméjean S. (2013). Minimal Intervention Dentistry: Part 5. Atraumatic Restorative Treatment (ART)—A Minimum Intervention and Minimally Invasive Approach for the Management of Dental Caries. *British Dental Journal*.

[128] Ericson D. (2004). What is Minimally Invasive Dentistry?. *Oral Health and Preventive Dentistry*.

[129] Laske M., Opdam N. J. M., Bronkhorst E. M. (2019). Minimally Invasive Intervention for Primary Caries Lesions: Are Dentists Implementing This Concept?. *Caries Research*.

[130] (2017). MI Dentistry Handbook - A Comprehensive Guide to Treatment Plans and Practice Implementation of Minimum Intervention Dentistry. https://www.gc.dental/europe/sites/europe.gc.dental/files/products/downloads/.

[131] Fontanab S. T. M. (2009). Patient Caries Risk Assessment.. *Monographs in Oral Science*.

[132] Giacaman R. A., Muñoz-Sandoval C., Neuhaus K. W., Fontana M., Chałas R. (2018). Evidence- Based Strategies for the Minimally Invasive Treatment of Carious Lesions: Review of the Literature. *Advances in Clinical and Experimental Medicine*.

[133] Oliveira D. C., Warren J. J., Levy S. M., Kolker J., Qian F., Carey C. (2016). Acceptance of Minimally Invasive Dentistry Among US Dentists in Public Health Practices.. *Oral Health & Preventive Dentistry*.

